# MPI-GWAS: a supercomputing-aided permutation approach for genome-wide association studies

**DOI:** 10.5808/gi.22001

**Published:** 2022-03-31

**Authors:** Hyojung Paik, Yongseong Cho, Seong Beom Cho, Oh-Kyoung Kwon

**Affiliations:** 1 Division of Supercomputing, Center for Supercomputing Application and Research, Korea Institute of Science and Technology Information (KISTI), Daejeon 34141, Korea; 2 Department of Data and HPC Science, University of Science and Technology (UST), Dae-jeon, 34141, Korea; 3 Department of Bio-Medical Informatics, Gachon University College of Medicine, Incheon 21565, Korea

**Keywords:** genome-wide association study, message-passing interface, parallel computing, supercomputing

## Abstract

Permutation testing is a robust and popular approach for significance testing in genomic research that has the advantage of reducing inflated type 1 error rates; however, its computational cost is notorious in genome-wide association studies (GWAS). Here, we developed a supercomputing-aided approach to accelerate the permutation testing for GWAS, based on the message-passing interface (MPI) on parallel computing architecture. Our application, called MPI-GWAS, conducts MPI-based permutation testing using a parallel computing approach with our supercomputing system, Nurion (8,305 compute nodes, and 563,740 central processing units [CPUs]). For 10^7^ permutations of one locus in MPI-GWAS, it was calculated in 600 s using 2,720 CPU cores. For 10^7^ permutations of ~30,000–50,000 loci in over 7,000 subjects, the total elapsed time was ~4 days in the Nurion supercomputer. Thus, MPI-GWAS enables us to feasibly compute the permutation-based GWAS within a reason-able time by harnessing the power of parallel computing resources.

## Introduction

Permutation testing is a nonparametric randomization procedure that provides a robust and powerful method of testing statistical hypotheses. In the original form of the permutation test, the response is shuffled *n* times, and it is commonly used when the standard distributional assumptions are violated. Although this random permutation is an attractive method in genome-wide association studies (GWAS), there is little guidance to achieve calculation efficiency for adjusting p-values via N-permutations using computing architecture.

A GWAS is an approach used in genetics to associate specific genetic variations with a particular trait, such as hair color. For example, using a GWAS approach, Ozaki et al. [1] discovered that a single nucleotide polymorphism (SNP) associated to myocardial infarction. With larger data sets in plant breeding, GWAS becomes very powerful in analyzing genetic contributions for traits, such as weight of each grain in spring wheat [2]. However, control for multiple testing and repeated observation of longitudinal traits are known limitations of GWAS. Although the permutation test can adjust type 1 errors of p-values in multiple tests, adaptive permutation approaches have been suggested, due to the computational cost of permutation, as an alternative solution [3]. Here, we present a software to solve this computational burden in GWAS.

## Methods

**Fig. 1.**
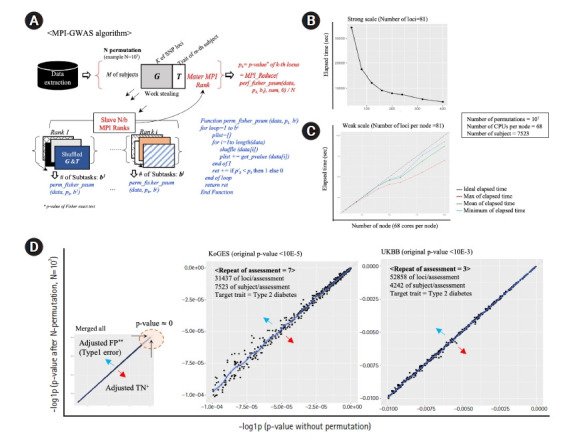
Algorithm overview and results. (A) Overview of the MPI-GWAS algorithm. (B) Performance of calculation parallelization of MPI-GWAS in the strong scale. (C) Performance of calculation parallelization of MPI-GWAS in the weak scale. (D) Analysis results of MPI-GWAS using the KoGES and the UKBB. MPI, message-passing interface; GWAS, genome-wide association study; CPU, central processing unit; KoGES, Korean Genome and Epidemiology Study; UKBB, UK biobank; FP, false-positive; TN, true-negative.

This note describes an open-source application, message-passing interface (MPI)-GWAS, which was designed for the adjustment of p-values of association analysis, such as Fisher’s exact test, of GWAS via N-permutation for each locus of the trait of interest. To optimize the time cost, a MPI-based work-stealing parallel scheme was applied, where MPI is a portable message-passing standard designed for development of parallel applications leveraging the power of manycore architecture at a supercomputer scale. Fig. 1A depicts the algorithm of MPI-GWAS. In a work-stealing scheme, each MPI process has a queue of computational tasks to perform. Each task fits models of variants by dividing by the number of permutations sequentially, but during its execution, a task may also spawn new tasks that can feasibly be executed in parallel. These new tasks are initially put in the queue. When an MPI task finishes work, it looks at the queues of the other MPI tasks and steals tasks. In effect, work stealing distributes the scheduled work over idle computer central processing units (CPUs), and while all CPU resources are being computed, scheduling overhead does not occur. Thus, the calculation time is greatly reduced. Moreover, MPI-GWAS effectively conducts permutation using Julia, an open-source project for high performance computing (https://julialang.org/). Using Julia, MPI-GWAS demonstrated a ~2–3-fold decrease in elapsed time of R.

The definition of the problem can be summarized into the following equations. We model the data of GWAS as follows.


(1) $$G=\left[\begin{array}{ccccc} g_{1,1} & \cdots & g_{1,k} & \cdots & g_{1,k}\\ g_{m,1} & \cdots & g_{m,k} & \ddots & \vdots \\ g_{M,1} & \cdots & g_{M,k} & \cdots & g_{M,k} \end{array}\right],\;T^{T}={\left[t_{1},t_{m},\cdots \;t_{M}\;\right]}^{T}$$

$$\text{where}\;{\text{g }}_{\text{(m,k)}}\text{=genotype}\;\text{of}\;\text{k-th}\;\text{locus}\;\text{in}\;\text{m-th}\;\text{subject}\;\text{(total}\;\text{subject=M),}\;{\text{t}}_{\text{m}\;}\text{=}\;\text{trait}\;\text{value}\;\text{of}\;\text{m-th}\;\text{subject}$$

Using the given observation, the genetic association between the k-th locus (i.e., k-th SNP) and Trait *T* is determined as $P^{T}={\left[p_{1\;}\cdots \;p_{k\;}\;\cdots \;p_{k\;}\right]}^{T}$. *P_k_* indicates the original p-value of Fisher’s exact test for the k-th locus. In general, the expected scale of *G* (i.e., number of SNPs) would be ~10^4^–10^n^. When the total number of permutations is N, MPI-GWAS shuffled the matrix *G* and *T* to generate background distributions of genetic diversity across *N/b* MPI ranks (where *b* = subtasks of shuffling numbers per MPI rank). Using these permutated matrices, each subtask per MPI rank calculates $P{'}^{T}={\left[p_{1}^{'}\;\cdots \;p_{k}^{'}\right]}^{T}$ as p-values of random distributions (Fig. 1A, blue pseudo-code). Thereafter, each MPI rank adds the result value of function *f* while looping the number of subtasks, where function *f* is defined as 1 if p'k < pk, otherwise 0; then, the master MPI rank yields the adjusted p-value of the k-th locus (*p"_k_*) by reducing the result value of other MPI tasks with the sum function and dividing by *N* (Fig. 1A, red pseudo-code). Specifically, *p'_k_* < *p_k_* indicates that the number of observations where random p-values of the k-th locus is less (i.e., more significant) than the calculated p-value using real observations. Therefore, the adjusted p-value means a probability of type 1 error occurrence for the k-th locus under N-time permutated distributions.

## Results

The strong and weak scaling performance of MPI-GWAS is presented in Fig. 1B and 1C, respectively. In summary, the strong scale (Fig. 1B) indicates that 10^7^ permutations of one locus can be calculated in 600 s using 2,720 CPU cores, which is 7.2 times faster than 272 cores. The weak scale (Fig. 1C) indicates that even if the number of permutations per one locus is increased according to the number of computation nodes, it performs well. Two cohorts of actual data were used to verify the performance of MPI-GWAS: the Korean Genome and Epidemiology Study (KoGES) [4] and the UK biobank (UKBB) [5]. The repeated observation of longitudinal traits, such as alteration of blood pressures along traced assessments for decades, is a representative example of the violation of the normal distribution of phenotypes. Thus, we utilized the traced phenotype of type 2 diabetes mellitus (T2DM) in the KoGES and UKBB, respectively. The phenotype of T2DM was measured repeatedly seven times every 2 years in the KoGES. Likewise, the participants of the UKBB have been assessed for the phenotype of T2DM up to three times across 10 years. The adjusted p-values via 10^7^ permutations using the KoGES and UKBB are displayed in Fig. 1D. In the case of the KoGES, covering 31,437 loci per assessment, a total computing time with 171,360 CPU cores was ~4 days using 2,500 nodes (25% of Nurion). With the UKBB data, covering 52,858 loci per assessment, the total elapsed time was similar. The selection of SNPs for KoGES is based on the traced loci using genotype array. To achieve a similar scale of validation, we used a subset of loci from the UKBB data. For the selection of 52,858 loci from the UKBB, we utilized the linkage disequilibrium pruning process via the PLINK. As depicted in Fig. 1D, type 1 errors of p-values were adjusted via large-scale N-permutations. In conclusion, MPI-GWAS enables us to feasibly compute the permutation-based GWAS within a reasonable time and to harness the power of supercomputing resources.

## Discussion

The parallel computing of MPI-GWAS solves the computational burden in the permutation approach for GWAS on an acceptable scale. To our best knowledge, MPI-GWAS is the first attempt for an acceleration of GWAS permutation using distributed memory system, including the Nurion. Moreover, the MPI is an interface standard for distributed memory parallelization. Although the partitioned global address space (PGAS) programming model has been suggested as the next step of MPI technology, practical usage of PGAS is still pending. The computing time of MPI-GWAS is mainly depending on the network bandwidth under shared memory system. For instance, the bandwidth of the Nurion is 100 GB/sec. Because we released our source code via GitHub for researchers (https://github.com/hypaik/proj_MPIGWAS), we expect the computing performance can be compared via many users. However, MPI-GWAS displays linearly improved performance with additional computing nodes. Because it was confirmed that the weak-scaling performance of MPI-GWAS comes close to ideal, it is predicted that it can perform well on large-scale cluster machines for calculations for more loci.

In addition, the utilized computing infra, Nurion system is a national supercomputing infra. Many computational research including astrophysics, nanoscience and protein docking simulations have been utilized this national supercomputing infra for over decades. The detail of web application for the use of the Nurion system is available at www.ksc.re.kr. Because we validated the performance of MPI-GWAS using the Nurion system, researchers can utilize directly to the Nurion system as well as other shared memory systems. In conclusion, our application could contribute broadly to GWAS globally, using diverse machines, including supercomputers.
